# Comparison of Ground-Based PM_2.5_ and PM_10_ Concentrations in China, India, and the U.S.

**DOI:** 10.3390/ijerph15071382

**Published:** 2018-07-02

**Authors:** Xingchuan Yang, Lei Jiang, Wenji Zhao, Qiulin Xiong, Wenhui Zhao, Xing Yan

**Affiliations:** 1 College of Resource Environment and Tourism, Capital Normal University, Beijing 100048, China; mx0yxc@163.com (X.Y.); xiong_ql@163.com (Q.X.); 2 Joint Center for Global Change Studies (JCGCS), Beijing 100875, China; 3 Beijing Municipal Research Institute of Environmental Protection, Beijing 100037, China; jiangle3657@sina.com; 4 Beijing Municipal Environmental Monitoring Center, Beijing 100048, China; Zhwenhui@163.com; 5 State Key Laboratory of Earth Surface Processes and Resource Ecology, College of Global Change and Earth System Science, Beijing Normal University, Beijing 100875, China

**Keywords:** particulate matter, spatiotemporal variations, air pollution, air quality index, environmental policy

## Abstract

Urbanization and industrialization have spurred air pollution, making it a global problem. An understanding of the spatiotemporal characteristics of PM_2.5_ and PM_10_ concentrations (particulate matter with an aerodynamic diameter of less than 2.5 μm and 10 μm, respectively) is necessary to mitigate air pollution. We compared the characteristics of PM_2.5_ and PM_10_ concentrations and their trends of China, India, and the U.S. from 2014 to 2017. Particulate matter levels were lowest in the U.S., while China showed higher concentrations, and India showed the highest. Interestingly, significant declines in PM_2.5_ and PM_10_ concentrations were found in some of the most polluted regions in China as well as the U.S. No comparable decline was observed in India. A strong seasonal trend was observed in China and India, with the highest values occurring in winter and the lowest in summer. The opposite trend was noted for the U.S. PM_2.5_ was highly correlated with PM_10_ for both China and India, but the correlation was poor for the U.S. With regard to reducing particulate matter pollutant concentrations, developing countries can learn from the experiences of developed nations and benefit by establishing and implementing joint regional air pollution control programs.

## 1. Introduction

Rapid economic development and increasing urbanization are partly responsible for rising particulate matter (PM) emissions. Exposure to particulate matter pollution has been associated with increased risks of respiratory, cardiovascular, cerebrovascular, and lung diseases [1,2,3]. According to the World Health Organization (WHO), air pollution has become a global environmental burden, with 92% of the world’s population currently living in areas where the air quality level exceeds the WHO guideline level of 10 μg m^−3^; about 3 million annual deaths worldwide are related to outdoor air pollution [4]. Assessing PM concentrations can improve our understanding of the effectiveness of pollution control and quantify its impacts on public health [5], regional visibility [6], and global climate change [7].

In order to prevent further worsening of air pollution, protect public health, and reduce economic losses, many countries have taken significant measures to improve air quality. Developed countries such as the U.S. have a long history of legislation against air pollution. In fact, the country is recognized as a global leader in mitigating air pollution. The U.S. Congress has passed and revised many laws in this regard, including the Air Pollution Control Act, the Clean Air Act, and the Motor Vehicle Air Pollution Control Act, which have laid the foundation for reducing emissions [8]. Although the air quality of most U.S. cities has now reached the recommended WHO level, PM pollution is still a significant concern in some western U.S. cities [9,10]. Moreover, developing countries such as China and India, which currently suffer from serious air pollution, are now beginning to establish and implement environmental management regulations and systems to control and mitigate air pollution. For example, the Chinese State Council and Ministry of Environmental Protection (MEP) promulgated “Ten Actions” in June 2013 and the Air Pollution Prevention Action Plan in September 2013. The urban air quality in the country has since improved [11]. The Indian government has also undertaken many steps, including announcing a National Clean Air Plan (NCAP), launching the National Air Quality Index, and banning the burning of biomass [12]. Although these measures have had some positive effects, more than 75% of Indian cities continue to report PM concentrations above the National Ambient Air Quality Standard. The matter is grave as air pollution reportedly causes about 1.1 million premature deaths each year in India alone [13]. Notably, the existing energy, industrial, and consumption structure of the Chinese and Indian economies cannot be completely reformed within a short period of time, and PM pollution management is a long-term task, particularly in economically developed metropolitan areas, which are likely to bear the brunt of air pollution. Therefore, PM pollution remains the focus of future air pollution prevention efforts in these countries.

Many recent studies have investigated the characteristics of PM pollution in different countries and regions. Previous studies on spatiotemporal PM variations have been based on either aerosol optical depth (AOD) data from ground monitoring [14], satellite observations [15], or visibility observations [16]. Indirect observation methods are often influenced by weather conditions and inversion models and contain large uncertainties. Thus, they cannot directly reflect air pollution status on the ground, leading to lower accuracy than ground monitoring. Ground monitoring provides direct observations of PM_2.5_ and PM_10_ concentrations (particulate matter with an aerodynamic diameter of less than 2.5 μm and 10 μm, respectively), which better reflect the true atmospheric environment. In China, for example, Wang et al. [17] systematically analyzed the spatiotemporal characteristics of six pollutants in 31 provincial capital cities from March 2013 to February 2014. They found that the PM_2.5_ and PM_10_ concentrations were higher in cities located in the northern region than those in the western and southeastern regions. Zheng et al. [11] studied the changes of PM_2.5_ emissions from 2013 to 2015 and demonstrated that PM_2.5_ concentrations in China showed a decreasing trend over the study period. Xie et al. [18] investigated the correlation between six pollutants in 31 provincial capital cities and found a strong correlation between PM_2.5_ and PM_10_. In India, the Indo-Gangetic Basin has been identified as having the most severe air pollution [19,20]. PM concentrations have been reported to be very high in Delhi [21], Raipur [22], Kolkata [23], and Kanpur [24]. In comparison, lower concentrations have been reported in Chennai [25] and Mumbai [26]. In recent years, there has been relatively little particulate contamination in the U.S. [27]. Thus, research in this area has been relatively scarce and the current studies mainly focus on areas where PM pollution continues to be relatively high, such as California [28] and Arizona [29]. However, as these studies were mainly concentrated in megacities, the results are fragmented and it is difficult to understand these trends from a macroscopic perspective. Further, although such studies systematically analyzed the temporal and spatial trends of PM concentrations, more in-depth research is needed on the relationships between different particle sizes. 

Few studies have presented inter-country comparisons—especially between developed and developing countries—of ground-based PM concentrations. Such comparisons are important as they can help developing countries draw lessons from the experiences of developed countries, and suitable targeted control measures can be planned and implemented accordingly. Thus, in this study, we examined the PM pollution characteristics for selected megacity regions in China, India, and the U.S. from 2014 to 2017 by analyzing the temporal and spatial variations in PM_2.5_ and PM_10_ concentrations and their trends.

## 2. Data and Methods

### 2.1. Data

**Figure 1:**
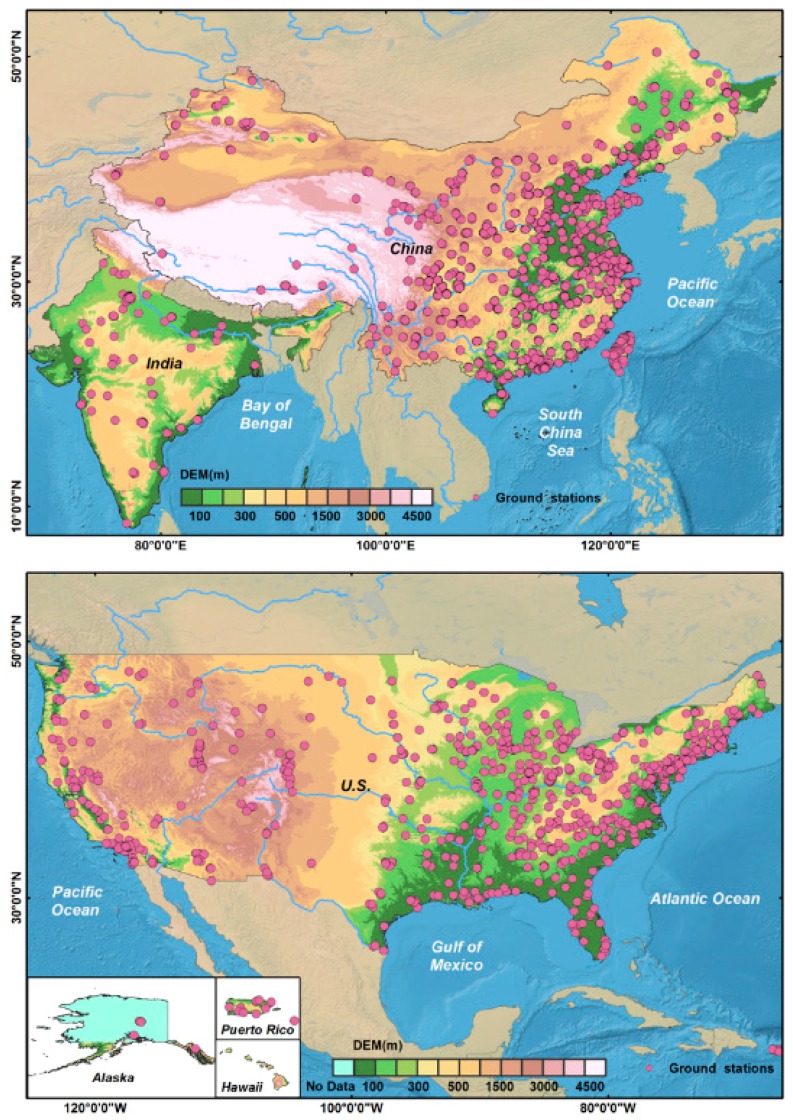
Particulate matter monitoring stations in China, India, and the U.S. used as data sources in this study.

We acquired PM data collected at monitoring stations in China, India, and the U.S. (Figure 1). A total of 1568 PM monitoring stations are distributed throughout China: 1471 in mainland China, 16 in Hong Kong, 5 in Macau, and 76 in Taiwan. The data were collected from the China Air Quality Real-time Distribution Platform (http://106.37.208.233:20035), the Hong Kong Environmental Protection Agency (https://cd.epic.epd.gov.hk/EPICDI/air/station/), the Macao Environmental Protection Agency (http://gis.dspa.gov.mo/AMEPB/AMEPBMainMap.aspx), and the Environmental Protection Agency of the Taiwan Executive Yuan (https://taqm.epa.gov.tw/taqm/en). India maintains 92 PM_2.5_ and 573 PM_10_ monitoring stations. The data were provided by the Central Pollution Control Department (CPCB) (http://www.cpcb.gov.in/CAAQM/Auth/frmViewReportNew.aspx). PM_2.5_ data availability was limited as the National Air Quality Monitoring Program (NAMP) of India mainly monitored sulfur dioxide (SO_2_), nitrogen dioxide (NO_2_), and PM_10_ concentrations before 2015. Therefore, we acquired PM_2.5_ data from January 2014 to December 2015 from 5 U.S. embassies in India (https://airnow.gov/index.cfm?action=airnow.global_summary). In the U.S., PM monitoring data were collected at 838 PM_2.5_ monitoring stations and 507 PM_10_ monitoring stations. These data were provided by the Environmental Protection Agency (https://aqs.epa.gov/aqsweb/airdata/download_files.html). More detailed information regarding data sources is given in Supplementary Table S1.

In order to ensure the integrity and representativeness of the evaluation, all the data were processed to reject spatial and temporal outliers. As each country maintains a different number of monitoring sites, the 24-h PM concentrations of all sites within that country were averaged to represent the overall PM pollution level. The total days of usable PM_2.5_ and PM_10_ data were 1461 for both concentrations for China, 1366 and 1434 respectively for India, and 1400 for both concentrations in the U.S.

### 2.2. Methods

To evaluate the air quality status with regard to PM in China, India, and the U.S., we analyzed four years (2014 to 2017) of ambient monitoring data for PM_2.5_ and PM_10_ in all three countries. To better understand each country’s spatial variations, 11 megacity regions were chosen for further analysis: Beijing-Tianjin-Hebei (BTH), the Yangtze River Delta (YRD), the Pearl River Delta (PRD), CY (Cheng-Yu), Delhi, Mumbai, Kolkata, Chennai, Hyderabad, San Diego-Los Angeles-San Francisco (SanSan), and Boston-NewYork-Washington (BosWash). More detailed information regarding megacity regions is given in supplementary Figure S1 and Table S2. These cities are the most economically developed and populated in their respective countries and represent different geographic and meteorological conditions. Pearson correlation analysis (r) was used as an indicator of the relationship between PM_2.5_ and PM_10_. The coefficient of variation (CV, namely, the standard deviation divided by the average) was used to describe the degree of spatial variation of PM concentrations in a given area, and it can be expressed as

(1) $$\mathrm{CV}=\mathrm{STD}/\bar{X}$$

Rate of change (ROC) was used to compare variance in PM_2.5_ and PM_10_ concentrations [30]:

ROC = (X − Y)/Y × 100%(2)

where Y represents the average PM_2.5_ or PM_10_ concentration in 2014, and X represents the average PM_2.5_ or PM_10_ concentration in a subsequent year.

## 3. Results

### 3.1. Temporal Variations

Table 1 and Table 2 summarize the characteristics of PM_2.5_ and PM_10_ concentrations, respectively, for the three countries from 2014 to 2017. These characteristics reflect the different attitudes of the country’s respective governments towards air quality (the annual data are presented in Supplementary Tables S3 and S4). PM levels were found to be lowest in the U.S., followed by China, and India. Although the mean PM_2.5_ and PM_10_ concentrations exceeded the median and showed a right-skewed distribution for all three countries, the U.S. showed lower PM concentrations and smaller standard deviations.

In the U.S., average daily concentrations of PM_2.5_ and PM_10_ ranged from 2.79–21.64 μg m^−3^ and 7.94–61.06 μg m^−3^, respectively. The respective average annual concentrations were 8.50 μg m^−3^ and 18.98 μg m^−3^, far below the WHO’s IT-1 standard (35 μg m^−3^ and 70 μg m^−3^, respectively). Additionally, PM_2.5_ (PM_10_) concentrations in the U.S. consistently decreased from 9.20 (19.54) μg m^−3^ to 7.94 (19.03) μg m^−3^ from 2014 to 2017. More than 98% (94%) of PM_2.5_ (PM_10_) concentrations in the U.S. met the IT-3 standard (i.e., 15 μg m^−3^ and 30 μg m^−3^, respectively) during the study period, demonstrating that PM pollution was rare. 

In China, average daily concentrations of PM_2.5_ and PM_10_ ranged from 6.03–126.03 μg m^−3^ and 15.58–217.04 μg m^−3^, respectively. The annual average concentrations of PM_2.5_ and PM_10_ were 1.28 and 1.12 times that of the WHO’s IT-1 standard. From 2014 to 2015, the concentrations of PM_2.5_ and PM_10_ increased by 5.33% and 6.19%, respectively, reflecting significant PM emissions. After the implementation of the Air Pollution Prevention Action Plan, the concentration of both pollutants decreased in subsequent years; for example, annual PM_2.5_ (PM_10_) concentrations decreased by 7.90% (2.58%) in 2015 compared to 2014. Although China’s annual average concentrations of PM_2.5_ (PM_10_) for all four studied years were still much higher than the IT-1 standard and 5–6 (4–5) times that of the U.S., the fraction of days meeting the standard increased from 30% in 2014 to 46% in 2017, reflecting improved air quality resulting from government measures to mitigate PM pollution.

In India, average daily concentrations of PM_2.5_ and PM_10_ ranged from 15.16–536.5 μg m^−3^ and 44.66–646.3 μg m^−3^, respectively. The corresponding average annual concentrations were 74.16 μg m^−3^ and 123.17 μg m^−3^, far above the IT-1 standard. The overall annual PM_2.5_ (PM_10_) concentrations in India were ~1.7 (~1.5) and ~8.7 (~6.4) times those of China and the U.S., respectively, with the lowest values being recorded in 2015 (PM_2.5_: 51.29 μg m^−3^; PM_10_: 99.83 μg m^−3^) and the highest in 2016 (PM_2.5_: 88.26 μg m^−3^; PM_10_: 155.58 μg m^−3^). Compared to 2014, the PM_2.5_ concentration in 2017 decreased by 9.50%, while PM_10_ increased by 31.90%. Furthermore, the fraction of days meeting the IT-1 standard remained low and hardly changed, suggesting continued emissions of PM pollutants [4]. 

**Figure 2:**
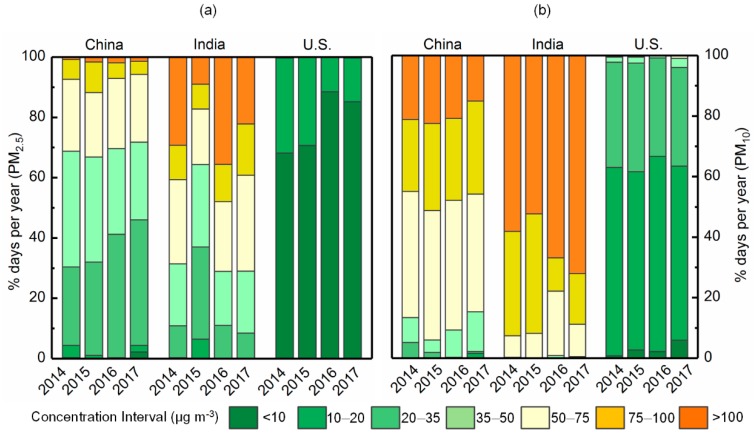
Annual range of daily particulate concentrations for China, India, and the U.S. for (**a**) PM_2.5_ and (**b**) PM_10_.

Figure 2 depicts the annual ratios of daily PM_2.5_ and PM_10_ concentrations in all three countries during the study period. In China, the daily concentrations of PM_2.5_ and PM_10_ mainly fell, ranging from 20–75 μg m^−3^ and 35–100 μg m^−3^, respectively. For PM_2.5_, the proportion of days with values of 20–35 μg m^−3^ increased from 26% (2014) to 42% (2017), while the proportion of days recording values above 100 μg m^−3^ decreased from 21% (2014) to 15% (2017). In India, PM_2.5_ concentrations exceeded 50 μg m^−3^ for a relatively high percentage of days (>69%), except in 2015 (36%). Most daily PM_10_ concentrations (%) exceeded 75 μg m^−3^. Furthermore, the number of days with PM_10_ concentrations greater than 100 μg m^−3^ increased from 2014 (58%) to 2017 (72%), indicating continued worsening of PM pollution in India. In the U.S., in contrast, most daily PM_2.5_ concentrations remained below 20 μg m^−3^ and those in the 0–10 μg m^−3^ interval increased from 68% (2014) to 89% (2017). Additionally, 90% of the daily PM_10_ concentrations were mainly distributed between 0 and 35 μg m^−3^. 

**Figure 3:**
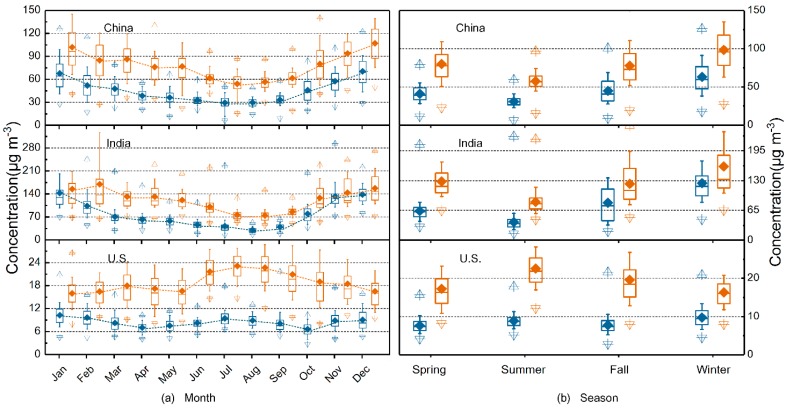
Particulate matter concentrations in China, India, and the U.S. from 2014 to 2017 by (**a**) month and (**b**) season. The blue and yellow box plots show seasonal 90th, 75th, 50th, 25th, and 10th percentiles of PM_2.5_ and PM_10_ concentrations, respectively; triangles indicate maximum and minimum values and diamonds indicate seasonal averages. Spring is defined as March through May, summer as June through August, fall as September through November, and winter as January, February, and December.

Figure 3 depicts monthly and seasonal variations of PM_2.5_ and PM_10_ concentrations in all three countries during the study period. China and India showed similar monthly patterns from January through December, depicted by a U-shaped curve. Both countries experience the rainy season from June to September, during which monthly PM values remained low and stable. From October through December, the onset of the dry season and increased residential heating resulted in an increasing trend, which then declined overall from January through May. In China, the most polluted month was December, with monthly PM_2.5_ (PM_10_) concentrations of 68.19 (107.22) μg m^−3^, 2.32 (1.98) times that of July’s lowest values. In India, PM_2.5_ and PM_10_ concentrations peaked in January (142.68 μg m^−3^) and December (157.04 μg m^−3^), respectively, while reaching their lowest level in June (30.80 μg m^−3^) and July (74.53 μg m^−3^). Compared with China and India, the monthly particulate concentrations in the U.S. were much lower, and showed less variation and a less consistent annual trend. PM_2.5_ and PM_10_ concentrations peaked in January (10.3 μg m^−3^) and July (23.14 μg m^−3^), respectively, reaching their lowest levels in October (6.64 μg m^−3^) and January (16 μg m^−3^). China and India showed similar seasonal patterns in which both PM_2.5_ and PM_10_ concentrations followed a clear annual cycle, with the highest values in winter and the lowest in summer. The average winter PM_2.5_ (PM_10_) concentrations in China and India were ~2.1 (~1.8) and ~2.3 (~1.9) times those for summer, respectively. This result is similar to the findings reported in previous studies [19,31]. In contrast, the U.S. followed a different pattern, in which both PM_2.5_ and PM_10_ concentrations were relatively high in the summer. 

### 3.2. Spatial Variations

**Figure 4:**
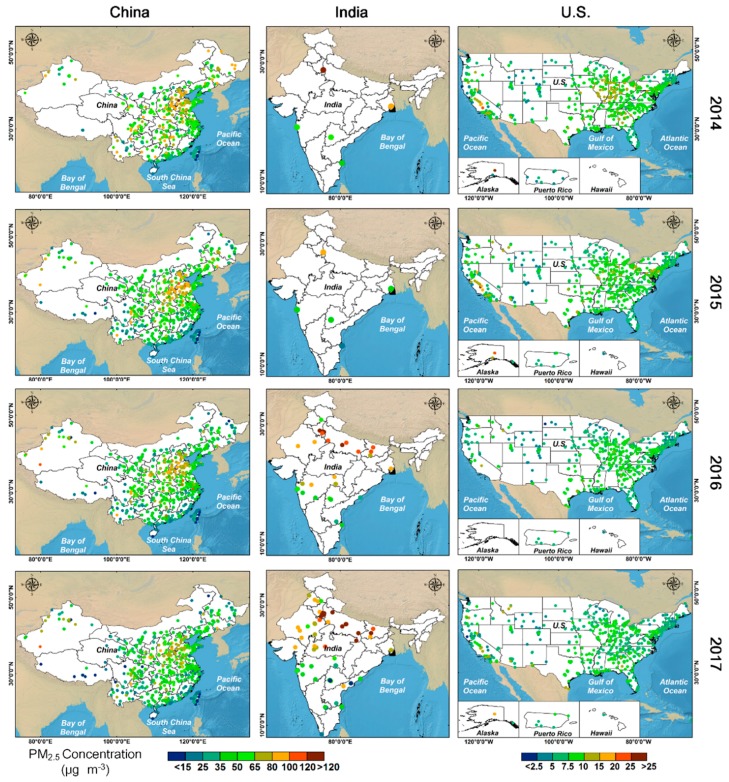
Spatial distribution of annual PM_2.5_ concentrations for China, India, and the U.S. from 2014 to 2017.

Figure 4 and Figure S2 show the spatial distributions of annual PM_2.5_ and PM_10_ concentrations, respectively, in China, India, and the U.S. The spatial patterns of PM_2.5_ and PM_10_ in China were similar, exhibiting a structure that is dispersed at large scales but aggregated at small scales. This result agrees with that of Cao et al. [32]. During the study period, higher PM_2.5_ and PM_10_ concentrations were generally observed in the North China Plain (e.g., BTH), the East China Plain (e.g., YRD), the Sichuan Basin (e.g., CY), and the Taklimakan Desert. Low PM_2.5_ and PM_10_ concentrations were found in the southwest (e.g., Tibet and Yunnan) and southeast (e.g., PRD, Hainan, and Taiwan). However, PM_10_ pollution was heavy in parts of the northwestern regions whereas the PM_2.5_ pollution was comparatively light. From 2014 to 2017, PM concentrations significantly declined in the most polluted Chinese cities. For example, in Beijing, the concentrations of PM_2.5_ (PM_10_) declined from 76.40 (113.59) μg m^−3^ to 56.86 (87.78) μg m^−3^. The number of moderately polluted cities also decreased significantly, but the number of cleaner cities remained relatively stable. However, PM levels in heavily polluted areas still exceeded China’s own standards and far exceed WHO standards.

In India, the spatial distributions of both PM_2.5_ and PM_10_ were highest in the north and lowest in the south, in agreement with the findings of Van et al. [33]. The regions with high PM_2.5_ and PM_10_ concentrations were mainly located in the Indo-Gangetic Plain; Delhi, Haryana, Uttar Pradesh, and Bihar are the most polluted states in India. PM concentrations were relatively low in most southern Indian states such as Kerala, Karnataka, and Tamil Nadu, which benefitted from the effects of land-sea breezes. From 2014 to 2017, the PM_2.5_ and PM_10_ concentrations in the most polluted cities continuously worsened due to rapid urbanization and industrialization and weak environmental enforcement [34]. However, most of southern India seemed to comply with the WHO’s IT-1 standard. 

During the study period, all of the stations in the U.S. had the annual PM_2.5_ and PM_10_ concentrations less than WHO’s IT-1 guideline, and most studied locations were below WHO’s IT-3 level. Higher concentrations were found along the west coast (e.g., SanSan and Alaska) and the central Midwest (e.g., Indiana and Illinois), while low concentrations were generally observed in most other parts of the U.S. This finding is in agreement with that of Van et al. [15]. From 2014 to 2017, the number of regions with relatively high PM_2.5_ and PM_10_ concentrations decreased substantially, although California continued to record higher levels of PM pollution. 

### 3.3. Correlation between PM_2.5_ and PM_10_ Concentrations

**Figure 5:**
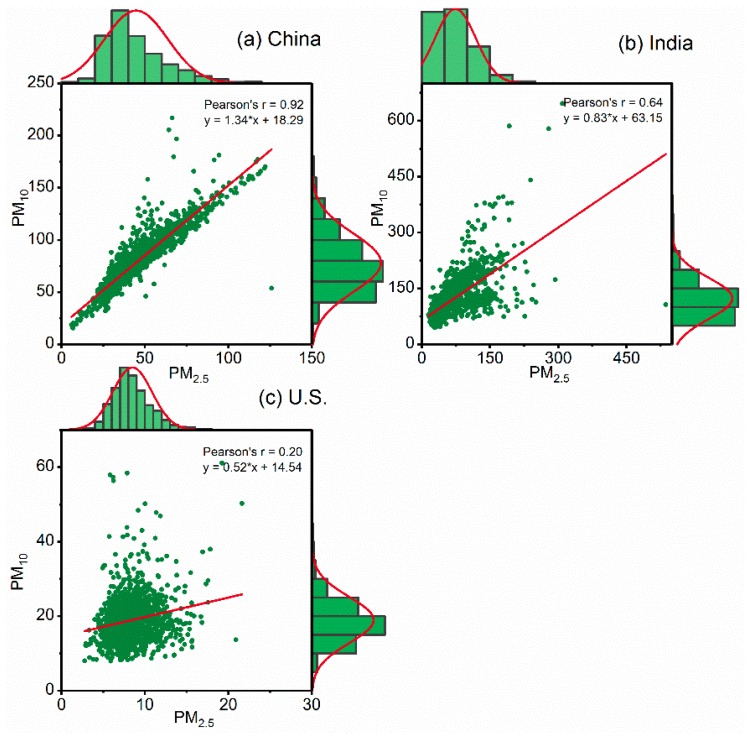
Correlation between the distributions of PM_2.5_ and PM_10_ for (**a**) China; (**b**) India; and the (**c**) U.S. from 2014 to 2017.

**Figure 6:**
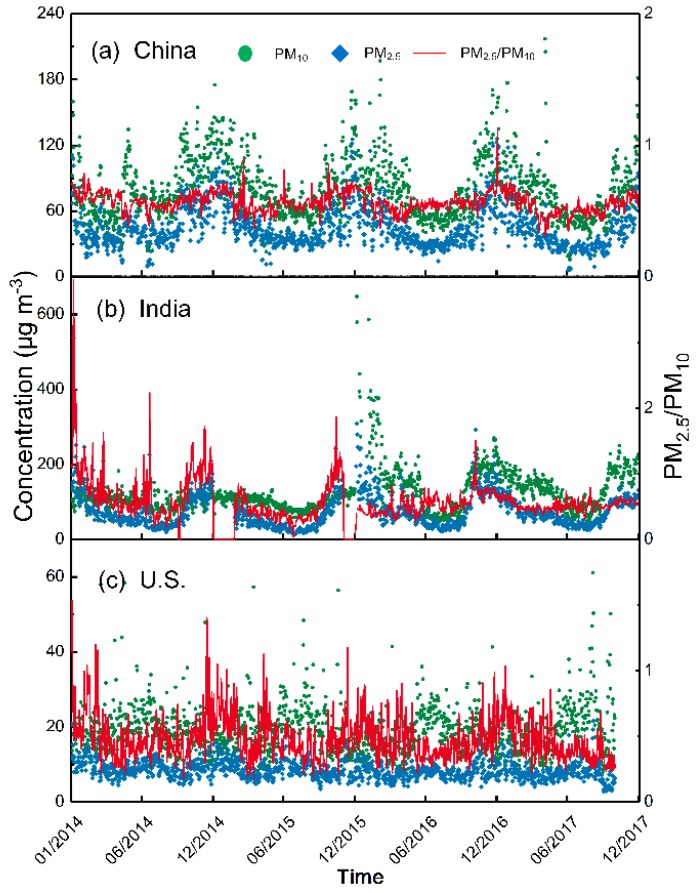
Daily concentrations and ratios of PM_2.5_ and PM_10_ in (**a**) China, (**b**) India and the (**c**) U.S. throughout the four years.

Correlations between changes in different particulate sizes in the same region are an important indicator of regional particulate pollution [35]. Therefore, we calculated the Pearson correlation coefficients (r) between PM_2.5_ and PM_10_ concentrations for all three countries over the study period (Figure 5). Daily PM_2.5_ and PM_10_ concentrations and ratios are presented in Figure 6. In China (Figure 5a), PM_2.5_ was highly correlated (r = 0.92) with PM_10_, similar to the correlation (0.94) found by Zhou et al. [36]. Approximately 62% of PM_2.5_ values were above the IT-1 standard as compared to 55.24% for PM_10_, suggesting that PM_2.5_ pollution was more serious. Furthermore, if the concentration of one pollutant reached a problematic level, the concentrations of the pollutant usually increased as well. For example, 53.32% of PM_10_ values exceeded this standard when PM_2.5_ concentrations exceeded the standard, while 54.21% of PM_2.5_ concentrations exceeded the standard when PM_10_ concentrations exceeded the standard (Figure 6a). As per Figure 5b, the correlation coefficient between PM_2.5_ and PM_10_ was 0.64 for India. It was noted that 84.40% of PM_2.5_ values were above the IT-1 standard as compared to 91.70% for PM_10_, suggesting that PM_10_ pollution was more serious. This result agrees with that of a Global Burden of Disease (GBD) report, which found that India’s PM_10_ pollution is more widespread compared to PM_2.5_ [13]. Furthermore, 78.83% of PM_10_ concentrations exceeded this standard when PM_2.5_ concentrations did not meet the standard, while 75.09% of PM_2.5_ concentrations exceeded the standard when PM_10_ concentrations did not comply with it (Figure 6b). The simultaneous variation in differently sized PM provides important evidence pertaining to the regional characteristics of PM pollution [35,37]. These results suggest that PM pollution in China and India have similar regional characteristics. Finally, extremely weak correlations (0.20) were detected between PM_2.5_ and PM_10_ for the U.S. (Figure 5c), indicating no obvious synchronous variation between the two concentrations.

### 3.4. PM_2.5_ and PM_10_ Concentrations in Megacity Regions

We compared PM concentrations in 11 megacity regions of China, India, and the U.S. (Figure S3). There were significant differences in PM concentrations, with the levels being generally much lower in the U.S. as it is more developed compared to cities in China and India. Delhi and Kolkata were the most polluted cities, followed by BTH, the YRD, and Mumbai, while the two megacity regions of SanSan and BosWash in the U.S. were the cleanest. Spatial variations in the annual average PM concentrations (Table 3) were largest in India (PM_2.5_: 0.61, PM_10_: 0.45), followed by China (PM_2.5_: 0.47, PM_10_: 0.36) and the U.S. (PM_2.5_: 0.28, PM_10_: 0.33). At the city level, the spatial variations of PM_2.5_ concentrations in Indian megacities were largest (>0.8), with Chennai reaching 1.41, and the PRD in China being the lowest (0.56). For PM_10_ concentrations, large spatial variations were observed in Kolkata, BTH, and Mumbai, while the spatial variations for the YRD and the PRD were smaller.

**Figure 7:**
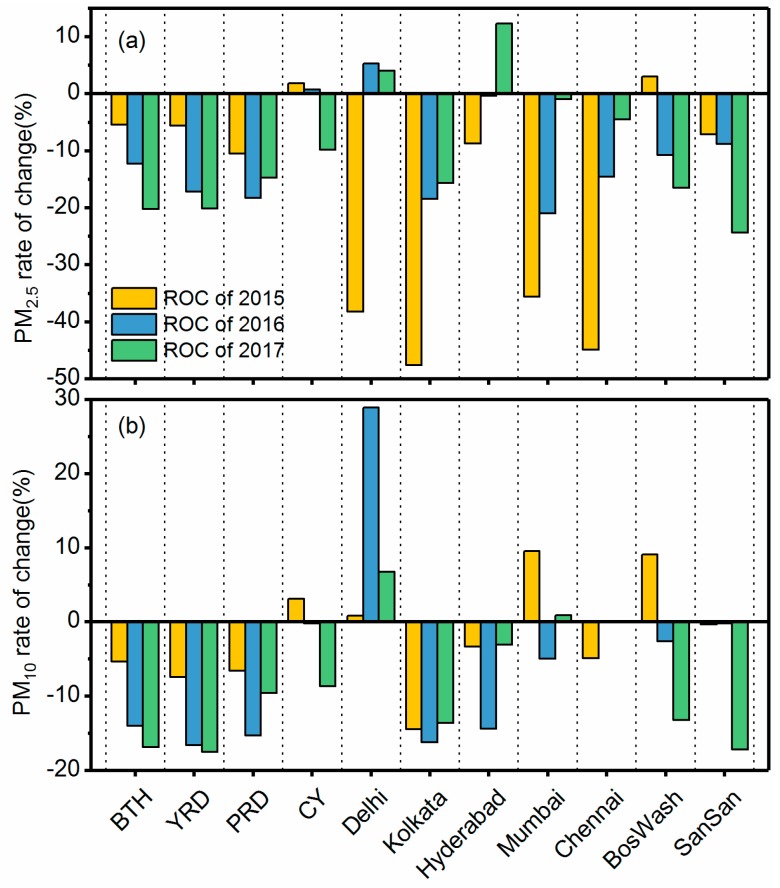
Rate of change for (**a**) PM_2.5_ and (**b**) PM_10_ from 2014 through the subsequent three years for 11 megacity regions in China, India, and the U.S.

Figure 7 shows the ROC in PM concentrations for three years following 2014; significant differences were found in each megacity region. In China, PM concentrations in the BTH, YRD, and PRD decreased every year, while PM concentrations in CY increased slightly in 2015 and 2016 before decreasing in 2017. Annual mean PM_2.5_ (PM_10_) concentrations in the BTH, YRD, PRD, and CY decreased from 2014 to 2017. The overall concentrations in all four regions generally declined, in agreement with Song et al. [38]. The 2015 concentrations of PM_2.5_ sharply decreased in four of the five Indian cities, with Hyderabad showing a smaller decrease. The concentrations of PM_2.5_ in Delhi and Hyderabad decreased in certain years, while they increased or remained stable for other years. Although the concentrations of PM_10_ in Kolkata and Hyderabad decreased in all three years, those in Delhi and Mumbai increased significantly in different years. The results suggest that further measures could be taken in India to control air pollution, especially in Delhi. In the U.S., PM concentrations mostly declined or remained stable, except for BosWash in 2015, which mirror’s the EPA’s report on national trends in PM_2.5_ and PM_10_ concentrations [39].

## 4. Discussion

This study investigated the spatial and temporal features of PM_2.5_ and PM_10_ concentrations in China, India, and the U.S. Developing countries suffer from serve PM pollution as they undergo rapid industrialization and urbanization. Most developed countries, such as the U.S., have already experienced this phase. The PM pollution can be attributed to increasing fossil fuel use by power-heavy industries, construction, vehicle exhaust, and biomass combustion [19,40,41]. China and India show significant seasonal variations in PM concentrations, the highest being in winter and the lowest in summer, while the opposite situation is noted for the U.S. This inconsistency is most likely due to the different seasonal variations of PM emissions and meteorological conditions in these countries [42,43]. For example, in China and India, the highest concentrations in winter coincide with massive emissions from fossil fuel and biomass burning for heating in winter [44,45,46]. Furthermore, more frequent occurrences of adverse meteorological conditions (e.g., slow winds, high humidity, and lower boundary layer height) may promote the accumulation of PM pollutants in these countries [47]. For the U.S., both PM_2.5_ and PM_10_ concentrations were relatively high in the summer, which can be attributed to the more frequent wildfire activity in this period [48]. In terms of spatial distribution, PM concentrations are generally higher in the north than in the south, and they are lower in the coastal regions compared to the inland regions in China and India. The most polluted regions were observed in the BTH and the Indo-Gangetic Plain. Similar conclusions have been reported in previous studies [33,44]. In the U.S., most cities, except for parts of the western regions [15], met the IT-1 standard for PM concentrations. This result indicates that different control strategies should be targeted for different regions, depending on regional emissions and meteorological characteristics [49]. Strong correlations between PM_2.5_ and PM_10_ concentrations were noted for China (0.92) and India (0.64), which could be explained by the similar sources of PM_2.5_ and PM_10_ pollutants, mainly emissions from combustion sources (e.g., coal, vehicle exhaust, and biomass) and dust [50,51]. However, weak correlations were detected in this regard for the U.S. (0.20). The main likely reason for this difference is that these pollutants arise from different sources. The latest version of the EPA’s National Emissions Inventory (NEI) concerning PM sourcing and elemental characteristics demonstrated that fires (i.e., wildfires, prescribed fires, and agricultural burning) are larger contributors to primary PM_2.5_ concentrations while dust (i.e., unpaved road dust, construction dust, and paved road dust) contributed largely to PM_10_ concentrations [52]. These results suggest that PM pollution in China and India has similar regional characteristics, and thus, regional collaboration in PM pollution control and mitigation could be an important approach to enhance urban and regional air quality. China has successful experiences in air pollution reduction due to its policy of regional collaboration. For example, with the establishment and optimization of the regional coordination mechanism for the BTH region since 2013, the air quality in this region has improved dramatically. Annual mean PM_2.5_ (PM_10_) concentrations of the BTH region decreased by 33% (34%) in 2016 compared to that of 2013 [53]. 

From 2014 to 2017, the PM_2.5_ and PM_10_ concentrations showed a decreasing trend in China and the U.S., and an increasing trend (albeit with some fluctuations) in India. Legislation plays a crucial role in air pollution management [54]. Table S5 lists the main legislations on air quality protection adopted by China, India, and the U.S. Many cities in the U.S. (e.g., Donora in Pennsylvania) experienced severe air pollution in the 1940s–1950s, causing serious health crises [55]. In response, the U.S. Congress passed the Clean Air Act and its subsequent amendments, and made remarkable progress in improving the nation’s air quality. This can be evidenced by the fact that the combined emissions of the six common pollutants (PM_2.5_ and PM_10_, SO_2_, NO_x_, volatile organic compounds, carbon monoxide, and lead) dropped by 73% from 1970 to 2016 [39]. China enacted the Air Pollution Prevention and Control Law in 1987, and amended it in 1995 and 2000. However, these laws were quite basic and enforcement was difficult due to inadequate financial and human resources. Thus, efforts to improve air quality in developing countries are typically ineffective [56]. Given the worsening air pollution and rising public concern, however, the Chinese government has successfully enacted more stringent and enforceable laws such as the Action Act in 2013 and the Air Pollution Prevention and Control Law in 2015. Thus, PM pollution across China, especially in its most polluted areas, has been significantly reduced [11,36]. However, in India, the laws regarding air pollution control (e.g., the Air (Prevention and Control of Pollution) Act) are outdated and weak. Moreover, while these laws cover the theoretical requirements for pollution control and mitigation, they have failed in terms of implementation [57]. Thus, PM pollution in India continues to be severe despite the enactment of various air pollution control regulations.

## 5. Conclusions

In this paper, we analyzed the spatiotemporal characteristics, change rates, and correlations of PM_2.5_ and PM_10_ values in China, India, and the U.S. Further, we discussed the main reason for the differences in PM pollution characteristics among the three countries and the impacts of their air pollution control laws on PM concentrations. Our main conclusions are as follows. PM levels in China and India were much worse than those in the U.S. from 2014 to 2017. A strong seasonal trend was observed in China and India, with the highest values occurring in winter and the lowest in summer. In contrast, PM pollutant distribution in the U.S. showed relatively high concentrations in summer and the relatively low values in winter. The most heavily polluted areas included the North China Plain and the Indo-Gangetic Plain, while less polluted areas included southwestern and southeastern China and southern India. PM concentrations in most of the studied regions in the U.S. met WHO standards; higher concentrations of PM_2.5_ and PM_10_ were found in California and the central Midwest. Although both PM_2.5_ and PM_10_ concentrations declined gradually in the U.S. and China—particularly in the severely polluted regions of China—during the study period, no comparable decrease occurred in India. In addition, PM_2.5_ and PM_10_ concentrations were highly correlated in both China and India but poorly correlated in the U.S. China and India still record high levels of PM pollution; however, both countries can learn from the experiences of the U.S. pertaining to air pollution laws and management. These results also suggest that regional joint prevention and control over air pollution is an important approach to improve the ambient air quality in cities and regions in China and India.

Our research contributes to the field as it increases understanding of the status and variations of PM_2.5_ and PM_10_ concentrations in China, India, and the U.S. However, our study also has several limitations. For example, the data used in this study were sourced from unevenly distributed ground stations in these countries. This poses a potential threat to the validity of the results. Moreover, long-term inter-country comparisons among countries are possible using satellite-derived PM data despite the uncertainties associated with them. Thus, in the future, more in-depth inter-nation comparisons should be made using satellite-derived PM concentrations in conjunction with ground-based measurements.

## Figures and Tables

**Table 1:** Summary of daily PM_2.5_ concentrations in China, India, and the U.S.

Statistic	China				India				U.S.			
	2014	2015	2016	2017	2014	2015	2016	2017	2014	2015	2016	2017
**Average PM_2.5_ Concentration (μg m^−3^)**												
Mean	45.19	47.60	44.74	41.62	79.70	51.29	88.26	72.13	9.20	8.85	7.92	7.94
Std. Dev.	17.69	19.75	18.79	19.45	49.91	34.00	52.82	28.98	2.32	2.45	1.89	2.50
Minimum	9.68	11.25	13.99	6.03	19.63	15.16	21.77	25.50	4.48	3.53	4.11	2.79
Median	41.57	41.30	38.52	37.61	63.79	39.66	71.95	67.30	8.95	8.50	7.76	7.67
Maximum	107.95	121.71	122.11	126.02	536.50	242.76	309.56	137.86	20.91	17.57	17.58	21.64
NOMs *	1041	1568	1568	1568	5	5	40	92	750	758	736	706
**Percentage of days attaining targets (%)**												
IT-1 (35 μg m^−3^) **	30	32	41	46	11	37	11	8	100	100	100	100
IT-2 (25 μg m^−3^) **	8	3	5	15	1	18	1	0	100	100	100	100
IT-3 (15 μg m^−3^) **	3	1	0	2	0	0	0	0	98	98	99	98

* Number of monitoring stations. ** WHO interim targets.

**Table 2:** Summary of daily PM_10_ concentrations in China, India, and the U.S.

Statistic	China				India				U.S.			
	2014	2015	2016	2017	2014	2015	2016	2017	2014	2015	2016	2017
**Average PM_10_ concentration (μg m^−3^)**												
Mean	77.08	81.85	78.75	75.10	103.35	99.83	155.58	136.31	19.54	19.28	18.08	19.03
Std. Dev.	27.16	27.27	27.71	28.76	20.11	17.74	88.68	46.28	5.87	6.34	5.10	7.47
Minimum	23.42	22.75	29.68	15.07	49.87	56.58	44.66	46.82	9.16	8.23	8.22	7.94
Median	72.58	76.00	72.41	71.37	102.65	101.48	152.24	139.43	18.70	18.24	17.40	17.95
Maximum	159.71	178.34	175.48	211.62	182.82	163.55	646.35	250.15	58.35	57.29	41.35	61.06
NOMs *	1041	1568	1568	1568	573	573	40	92	468	429	412	378
**Percentage of days attaining targets (%)**												
IT-1 (70 μg m^−3^) **	44	41	46	48	2	4	19	9	100	100	100	100
IT-2 (50 μg m^−3^) **	13	6	9	15	0	0	1	1	99	99	100	99
IT-3 (30 μg m^−3^) **	3	1	0	2	0	0	0	0	96	95	98	94

* Number of monitoring stations. ** WHO interim targets.

**Table 3:** Coefficients of variation (CV) of PM_10_ and PM_2.5_ in China, India, and the U.S.

	PM_2.5_					PM_10_				
	2014	2015	2016	2017	Average	2014	2015	2016	2017	Average
**China**	0.39	0.42	0.42	0.47	0.43	0.35	0.33	0.35	0.38	0.36
BTH	0.82	0.87	0.91	0.85	0.72	0.67	0.70	0.74	0.75	0.87
CY	0.59	0.69	0.62	0.78	0.61	0.54	0.60	0.57	0.68	0.68
PRD	0.59	0.63	0.59	0.62	0.56	0.53	0.56	0.56	0.58	0.61
YRD	0.54	0.64	0.65	0.64	0.57	0.54	0.56	0.59	0.56	0.63
**India**	0.63	0.66	0.60	0.40	0.61	0.19	0.18	0.57	0.34	0.45
Chennai	0.59	0.71	0.80	1.49	1.41	0.49	0.61	*	*	0.55
Delhi	0.90	0.99	0.98	0.96	0.98	0.52	0.48	0.69	0.76	0.73
Hyderabad	0.61	0.60	0.68	0.89	0.81	0.43	0.45	0.42	0.71	0.67
Kolkata	0.91	1.26	0.95	0.90	1.00	0.65	0.63	1.05	0.85	0.93
Mumbai	0.72	1.11	1.03	0.97	0.99	0.59	0.64	0.87	0.78	0.82
**U.S.**	0.25	0.28	0.24	0.32	0.28	0.30	0.33	0.28	0.39	0.33
BosWash	0.58	0.63	0.56	0.58	0.60	0.66	0.64	0.71	0.61	0.67
SanSan	0.87	0.76	0.64	0.59	0.75	0.90	0.80	0.73	0.68	0.81

* No data were available.

## Supplementary Materials

The following are available online at http://www.mdpi.com/1660-4601/15/7/1382/s1, Table S1: Summary of datasets applied in this study, Table S2: Basic information of megacity regions in China, India, and the U.S., Table S3: Summary of PM_2.5_ concentrations in China, India, and the U.S., Table S4: Summary of PM_10_ concentrations in China, India, and the U.S., Table S5: Main legislations on air protection adopted in China, India, and the U.S., Figure S1: Locations of megacity regions in China, India, and the U.S., Figure S2: Spatial distribution of PM_10_ annual average concentrations of provinces (states) in China, India, and the U.S. from 2014 to 2017, Figure S3: Yearly distribution for PM concentrations in megacity regions in China, India, and the U.S. from 2014 to 2017.
