# Supplementary material for: Comparison of Ground-Based PM2.5 and PM10 Concentrations in China, India, and the U.S
Source: Int J Environ Res Public Health. 2018 Jul 2;15(7):1382. doi: 10.3390/ijerph15071382 (PMC6068888; doi:10.3390/ijerph15071382)
Supplement: Supplementary file 1 [file ijerph-15-01382-s001.pdf]

Supplementary Materials

# Comparison of Ground-Based $PM_{2.5}$ and $PM_{10}$ Concentrations in China, India, and the U.S.

**Xingchuan Yang, Lei Jiang, Wenji Zhao, Qiulin Xiong, Wenhui Zhao, Xing Yan**

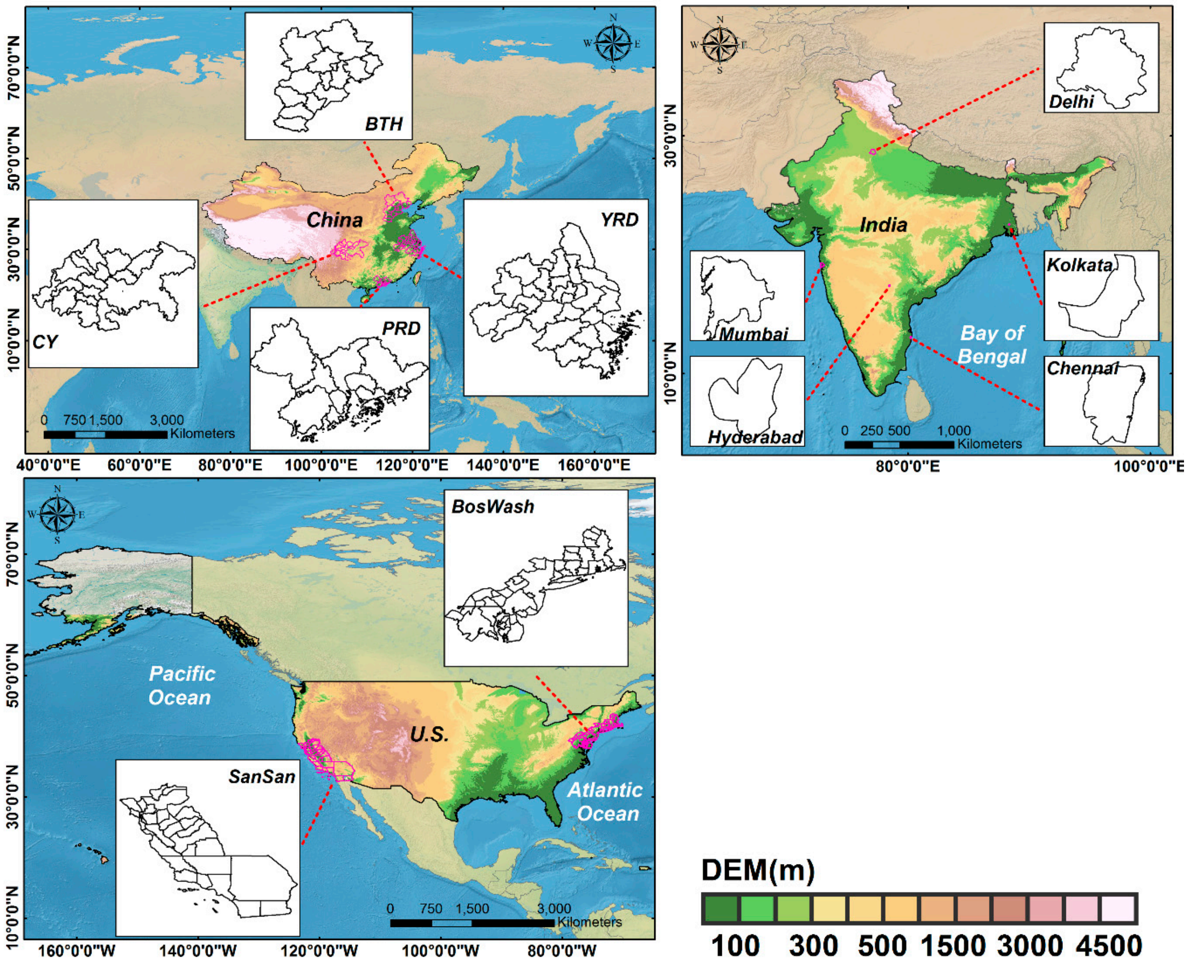


**Figure S1.** Locations of megacity regions in China, India and the U.S.

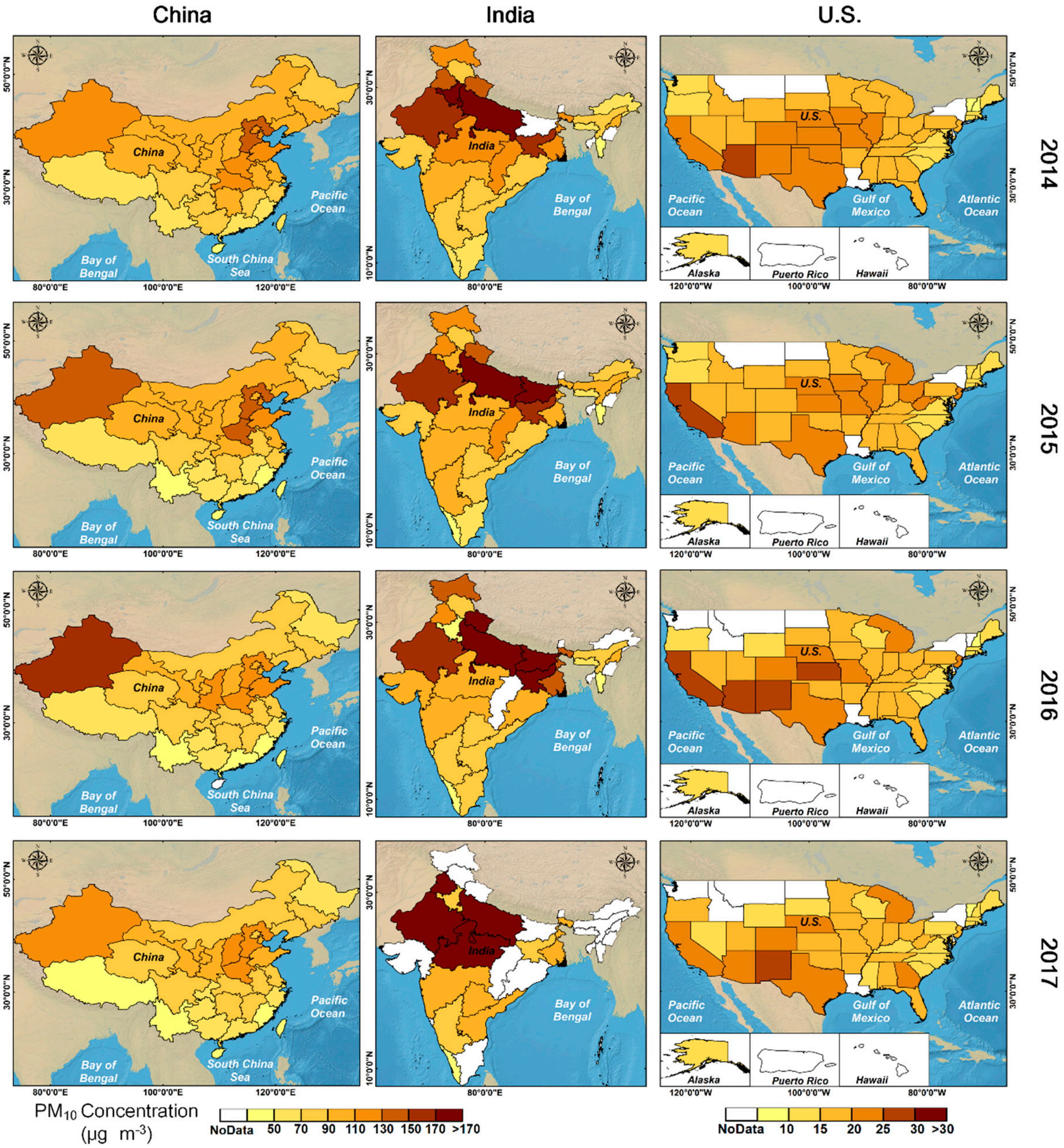


**Figure S2.** Spatial distribution of $PM_{10}$ annual average concentrations of provinces (states) in China, India, and the U.S. from 2014 to 2017.

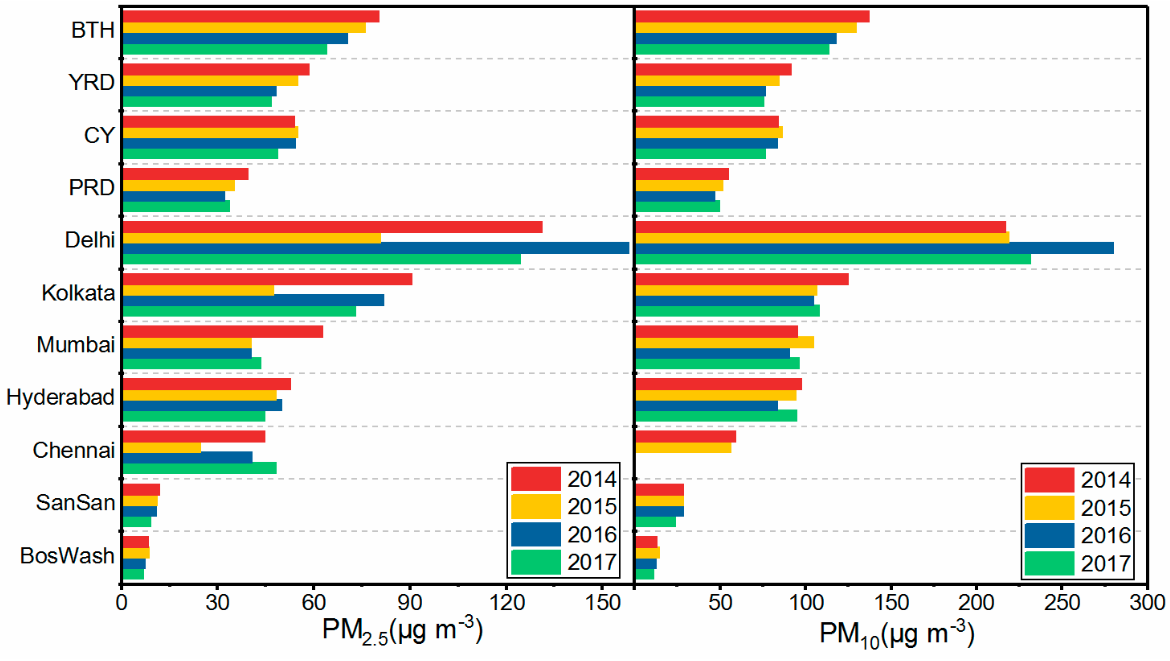


**Figure S3.** Yearly distribution for PM concentrations in megacity regions in China, India, and the U.S. from 2014 to 2017.

**Table S1.** Summary of datasets applied in this study.

| Country | Region | Particulate matter | Source | Provider |
|---|---|---|---|---|
| **China** | Mainland | $PM_{2.5}$;$PM_{10}$ | National Urban Air Quality Real-time Publishing Platform | China National Environmental Monitoring Centre |
| | Hong Kong | | data.gov.hk | Hong Kong Environmental Protection Department |
| | Macao | | Environmental Geographic Information System | Macao Meteorological and Geophysical Bureau |
| | Taiwan | | Taiwan Air Quality Monitoring Network | Environmental Protection Administration, Executive Yuan |
| **India** | / | $PM_{2.5}$ | AirNow Department of State(2014-2015); Central Control Room for Air Quality(2016-2017) | Central Pollution Control Board |
| | | $PM_{10}$ | data.gov.in(2014-2015);Central Control Room for Air Quality(2016-2017) | |
| **U.S.** | / | $PM_{2.5}$;$PM_{10}$ | AirNow | United States Environmental Protection Agency |

**Table S2.** Geographic information of megacity regions in China, India, and the U.S.

| Countries | Regions | Geomorphic type | Climate type |
|---|---|---|---|
| **China** | BTH | North China Plain | Temperate continental climate |
| | CY | Sichuan Basin | Subtropical monsoon climate |
| | PRD | Pearl River Delta Plain | Subtropical monsoon climate |
| | YRD | Middle-Lower Yangtze plains | Subtropical monsoon climate |
| **India** | Chennai | Chennai Basin | Tropical monsoon climate |
| | Delhi | Gangetic Plain | Tropical monsoon climate |
| | Hyderabad | Deccan Plateau | Tropical monsoon climate |
| | Kolkata | Gangetic Plain | Tropical monsoon climate |
| | Mumbai | coastal plain | Tropical monsoon climate |
| **U.S.** | BosWash | Atlantic Coastal Plain | Subtropical humid climate; Temperate continental climate |
| | SanSan | Central Valley | Mediterranean climate |

**Table S3.** Summary of $PM_{2.5}$ concentrations in China, India, and the U.S.

| Statistic(2014-2017) | $PM_{2.5}$(μg $m^{-3}$) | | | | | |
|---|---|---|---|---|---|---|
| | **Mean** | **Minimum** | **Median** | **Maximum** | **St.Dev.** | **ROC(%)** |
| **China** | 44.79 | 6.03 | 39.71 | 126.03 | 19.06 | -7.90 |
| **India** | 74.16 | 15.16 | 62.17 | 536.5 | 45.03 | -9.50 |
| **U.S.** | 8.50 | 2.79 | 8.19 | 21.64 | 2.36 | -13.62 |

**Table S4.** Summary of $PM_{10}$ concentrations in China, India, and the U.S.

| Statistic(2014-2017) | $PM_{10}$(μg $m^{-3}$) | | | | | |
|---|---|---|---|---|---|---|
| | **Mean** | **Minimum** | **Median** | **Maximum** | **St.Dev.** | **ROC(%)** |
| **China** | 78.21 | 15.58 | 73.22 | 217.04 | 27.81 | -2.58 |
| **India** | 123.17 | 44.66 | 110.16 | 646.35 | 55.82 | 31.90 |
| **U.S.** | 18.98 | 7.94 | 18.24 | 61.06 | 6.22 | -2.56 |

**Table S5.** Main legislations on air protection adopted in China, India, and the U.S.

| U.S. | China | India |
|---|---|---|
| **1955** Air Pollution Control Act | | |
| **1963** Clean Air Act | | |
| **1965** Motor Vehicle Air Pollution Control Act | | |
| **1967** Air Quality Act | | |
| **1970** Clean Air Act Amendments of 1970 | | |
| **1977** Clean Air Act Amendments of 1977 | | |
| | | **1982** The Air (Prevention and Control of Pollution) Rules |
| | | **1983** The Air (Prevention and Control of Pollution) (Union Territories) Rules, 1983 |
| | **1987** Law on the Prevention and Control of Atmospheric Pollution | **1987** The Air (Prevention and Control of Pollution) Act 1981,amended 1987 |
| **1990** Clean Air Act Amendments of 1990 | | |
| | **1995** Law on the Prevention and Control of Atmospheric Pollution(revised in 1995) | |
| | **2000** Law on the Prevention and Control of Atmospheric Pollution(amended in 2000) | |
| | **2003** Environmental Impact Assessment Law | |
| **2009** The American Clean Energy and Security Act of 2009 | | |
| | | **2010** The National Green Tribunal Act |
| **2011** Cross-State Air Pollution Rule | | |
| | **2012** Law on Promoting Clean Production | |
| | **2013** Air Pollution Prevention Action Plan | |
| | **2015** Environmental Protection Law; Air Pollution Prevention and Control Law | |
